# Initial presentation, etiology and risk factors for adverse outcomes in infection-associated plastic bronchitis in children: a retrospective study

**DOI:** 10.3389/fped.2026.1740626

**Published:** 2026-03-13

**Authors:** Huan Cao, Dongge Liang, Han Huang, Qianqian He, Linlin Wu

**Affiliations:** Respiratory Department, Children’s Hospital Affiliated to Zhengzhou University, Henan Children’s Hospital, Zhengzhou Children’s Hospital, Zhengzhou, China

**Keywords:** etiology, imaging features, mycoplasma pneumoniae, outcomes, plastic bronchitis

## Abstract

**Objective:**

To summarize the pathogen distribution, imaging features, radiologic outcomes, and risk factors for poor prognosis in children with infection-associated plastic bronchitis (PB).

**Methods:**

This retrospective study included 86 hospitalized children diagnosed with infection-associated PB at Henan Children's Hospital from January 2021 to December 2023. Patients were grouped based on radiologic findings and outcomes into favorable prognosis, poor prognosis, and indeterminate outcome groups. Clinical data and laboratory results were compared between the favorable and poor prognosis groups to identify potential risk factors for adverse outcomes.

**Results:**

Among 86 children with infection-associated PB, 81 (94.2%) were caused by Mycoplasma pneumoniae (MP), including 29 (35.8%) co-infections. Of the MP-associated cases, 65 (80.2%) showed a good outcome, and 10 (12.3%) had unclear outcomes, while 6 (7.4%) developed adverse outcomes. These included 1 case requiring surgery, 3 with persistent atelectasis, 1 with both atelectasis and bronchiectasis, and 1 with persistent bronchiectasis. Patients with adverse outcomes exhibited significantly higher levels of LDH (788.5 U/L vs. 408 U/L, *p* = 0.01) and D-dimer (2.20 μg/mL vs. 0.81 μg/mL, *p* = 0.01) compared to the good-outcome group. The adverse-outcome group also required more bronchoscopic interventions (median 3.5 vs. 2, *p* < 0.01), higher glucocorticoid doses (methylprednisolone 20 mg/kg/day vs. 5 mg/kg/day, *p* = 0.01), and more frequent use of low molecular weight heparin (100% vs. 21.5%, *p* < 0.01) and intravenous immunoglobulin (50% vs. 9.2%, *p* = 0.02).

**Conclusion:**

Mycoplasma pneumoniae is the predominant pathogen in infection-associated PB. All patients with poor outcomes were infected with M. pneumoniae. Although most children responded well to treatment, a subset developed long-term complications such as bronchiectasis and atelectasis, even requiring lobectomy. Elevated LDH and D-dimer levels may serve as early biomarkers for predicting unfavorable outcomes.

## Introduction

Plastic bronchitis (PB) is a rare condition characterized by the formation of branching mucoid bronchial casts that obstruct the tracheobronchial tree. While PB is known to be associated with various underlying conditions, including congenital heart disease, asthma, cystic fibrosis, and pulmonary infections, research on infection-associated PB remains limited despite recent increases in studies on this topic (1). Previous studies on infection-related plastic bronchitis have primarily concentrated on the investigation of etiological factors and the identification of risk factors contributing to cast formation (2–8). However, there has been no focused investigation into the outcomes of infection-associated PB. This study aims to fill this critical gap by conducting a retrospective study of 86 pediatric patients diagnosed with infection-related PB, examining the etiology, imaging characteristics, radiographic outcomes, and risk factors for adverse outcomes.

## Methods

This study retrospectively reviews patients with PB who were admitted to Henan Children's Hospital from January 2021 to December 2023, as retrieved from the hospital information system (HIS). Patients were included in the study based on the following criteria: the presence of recent new lesions confirmed by chest imaging, clear and reliable evidence of the causative pathogen, and the visibility of bronchial casts on bronchoscopy. Patients with underlying conditions that may influence the development of plastic bronchitis, such as allergic bronchopulmonary aspergillosis, immunodeficiency, or primary ciliary dyskinesia, were excluded.

The pathogen distribution and diagnostic methods indicated in each case record were analyzed. The cases were divided into two groups based on the presence or absence of infection with Mycoplasma pneumoniae (Mp). The chest imaging data of each case were reviewed to assess the radiographic findings, any comorbidities, disease progression, and outcomes in both groups. All patients in the non-Mp group demonstrated favorable prognosis. Within the group with Mp, further subdivision was made based on imaging characteristics and outcomes into three categories: those with a good outcome, those with an adverse outcome, and those who were unreachable or had an unclear long-term outcome. To date, there have been no studies on the outcomes of plastic bronchitis. Therefore, in defining the criteria for poor outcomes in this study, we primarily referenced previous research on the sequelae of community-acquired pneumonia (9), particularly those related to Mycoplasma pneumoniae pneumonia (10). Ultimately, based on our research, the following criteria were established for adverse outcomes: requiring surgical intervention due to pulmonary lesions or those with persistent complications after six months, such as bronchial obstruction, atelectasis, and poor absorption of necrotizing pneumonia. The group with a good outcome had complete or substantial absorption of lung infection without long-term complications. The group of an unclear outcomes comprised cases in which follow-up chest imaging was not conducted, rendering evaluation impossible. It also included cases where complications such as necrotizing pneumonia, bronchiectasis, or atelectasis were observed on imaging, but the follow-up period was less than six months, preventing a definitive assessment of long-term outcomes.

Data were collected from patients with Mp associated PB (Mp-PB), including age, gender, height, weight, clinical symptoms, white blood cell count (WBC), C-reactive protein (CRP), procalcitonin (PCT), lactate dehydrogenase (LDH), D-dimer, interleukin-6 (IL-6), Immunoglobulin E (IgE), abnormal liver function (ALT or AST > 80 U/L), time of first bronchoscopy (Time-B), number of bronchoscopy interventions (Num-B), time of first administration with methylprednisolone (Time-M), maximum dose of methylprednisolone (Max-M), choice of antibiotic agents and whether to administer second-line drugs including doxycycline and levofloxacin, and other treatments (intravenous immunoglobulin, thoracentesis drainage, anticoagulant therapy, oxygen therapy, surgery, etc.). For test indicators, if multiple tests were conducted, the first result after onset was included. Statistical analysis were performed by SPSS Statistical software v21. Continuous variables following a normal distribution are described as the mean ± standard deviation, while those not adhering to a normal distribution are presented as the interquartile range. Categorical variables are expressed as percentages. Fisher's exact test was used for categorical variables and *t*-tests or Mann–Whitney-test for continuous variables. A *P*-value of <0.05 was considered statistically significant.

**Figure 1:**
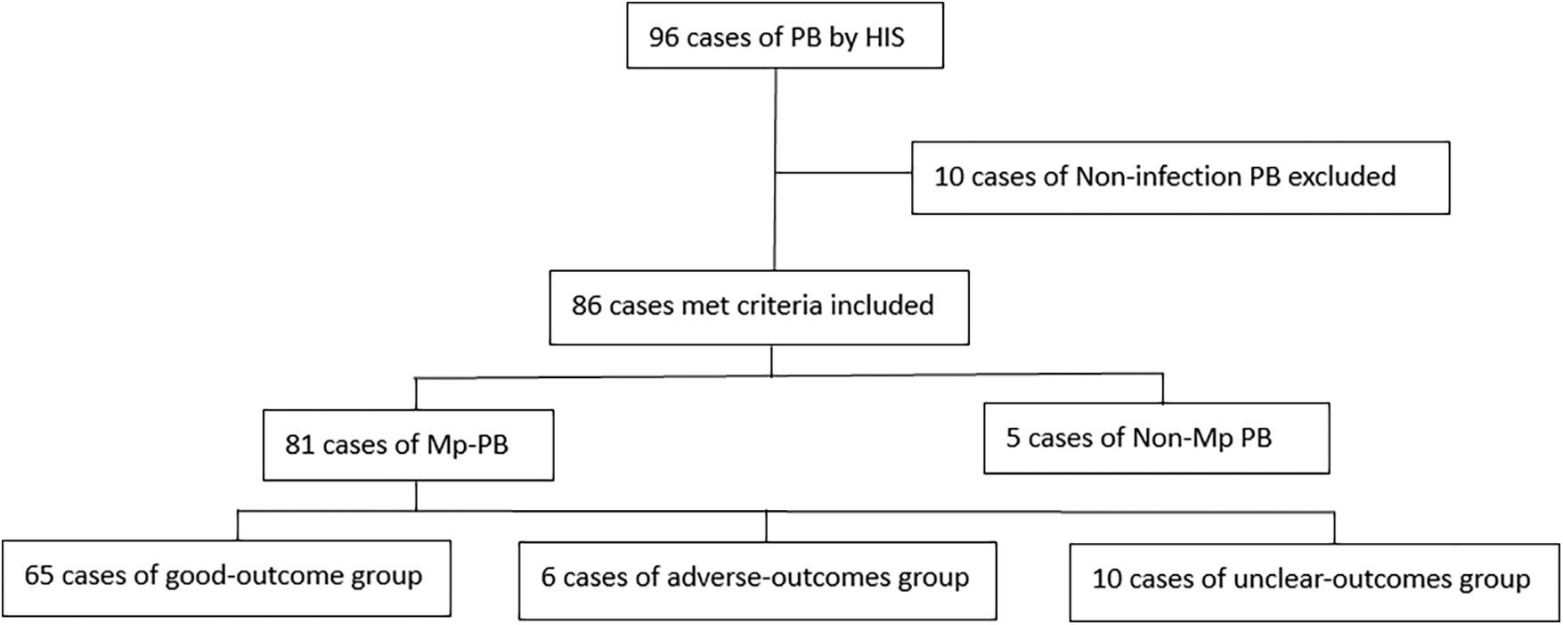
Flow chart of research. PB, plastic bronchitis; HIS, hospital information system; Mp, mycoplasma pneumoniae.

The research procedure is outlined in the flowchart (Figure 1).

## Results

We retrospectively reviewed 96 cases of PB identified via the HIS, of which 86 met the inclusion criteria. During the same period, a total of 36,916 patients were hospitalized due to pulmonary infection, among whom 86 cases were associated with PB, yielding an incidence rate of only 0.2%. Of these, 12,108 were diagnosed with *Mycoplasma pneumoniae* pneumonia (MPP), and 81 of them also had PB, resulting in an incidence rate of 0.7%.

### Pathogens

Among the 86 cases, 81 involved Mp, including 52 cases of isolated Mp and 29 cases of co-infections. The co-infections included Haemophilus influenzae type B (Hib, *n* = 8), Streptococcus pneumoniae (Sp, *n* = 7), Staphylococcus aureus (Sa, *n* = 2), adenovirus (*n* = 5), rhinovirus (*n* = 4), parainfluenza virus (*n* = 2), and respiratory syncytial virus (*n* = 1). There were 5 non-Mp infections: 2 cases of influenza A (A selected case is illustrated in Supplementary Figure S1), one co-infected with Sp and another with Acinetobacter baumannii; and 3 additional cases, including 1 of Hib co-infected with bocavirus, 1 of Sp, and one of parainfluenza virus type 3 co-infected with human respiratory virus type 3.

Among the 81 Mp associated PB (Mp-PB), 40 were definitively diagnosed via PCR (DNA or RNA) of sputum or bronchoalveolar lavage fluid (BALF), 11 through Mp antibody titer of serum of IgM, 25 via a combination of Mp antibody titer and PCR, and 5 through targeted Next-Generation Sequencing of BALF.

The A2063G mutation site of the macrolide resistance gene in Mp was analyzed in 47 patients. The mutation rates in the good, adverse, and unclear outcomes groups were 79% (30/38), 100% (4/4), and 100% (5/5), respectively, showing no significant statistical difference.

The results of pathogens analysis are presented in Table 1.

**Table 1:** Pathogens detected of 86 pediatric cases with infection associated PB.

Pathogens	*n* (%)
MP	81 (94.2)
Single Mp	52 (60.5)
Co-infection	29 (33.7)
Hib	8 (9.3)
Sp	7 (8.1)
Sa	2 (2.3)
Adenovirus	5 (5.8)
Rhinovirus	4 (4.7)
PIV	2 (2.3)
RSV	1 (1.1)
Non-MP	5 (6)
Influenza A	2 (2.3)
Co-infection with Sp	1 (1.1)
Co-infection with Ab	1 (1.1)
Hib + Bocavirus	1 (1.1)
Sp	1 (1.1)
PIV3 + HRV3	1 (1.1)

Mp, mycoplasma pneumoniae; Hib, haemophilus influenzae type B; Sp, streptococcus pneumoniae; Sa, staphylococcus aureus; PIV, parainfluenza virus; RSV, respiratory syncytial virus; Ab, acinetobacter baumannii; HRV3, human respiratory virus type 3.

### Radiographic manifestations, comorbidities, and outcomes

**Figure 2:**
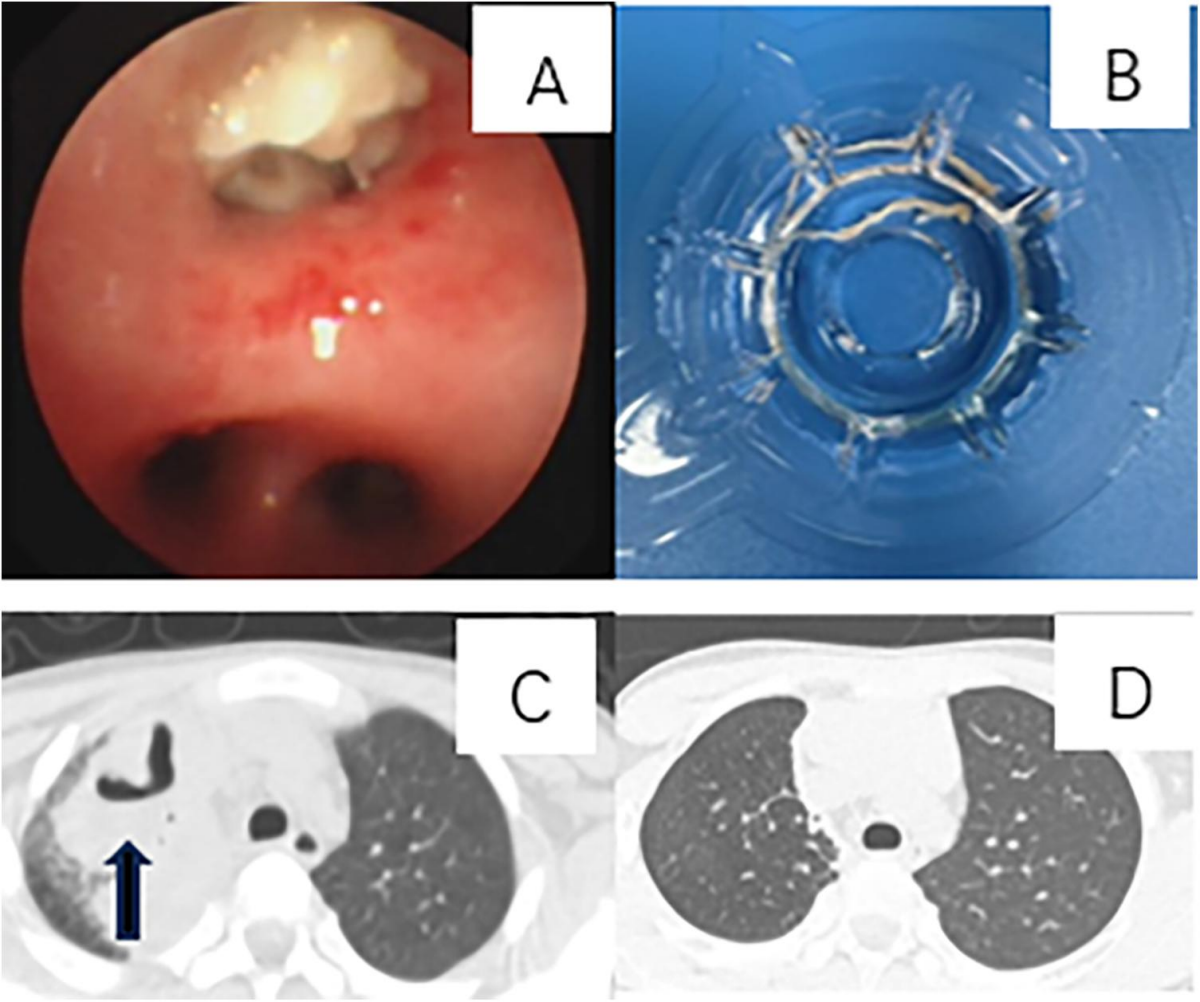
A case of plastic bronchitis associated with Mycoplasma pneumoniae infection presenting with temporary necrotizing pneumonia and subsequent complete recovery. A 10-year-and-3-month-old boy was diagnosed with Mp infection. He underwent bronchoscopy interventions on days 10, 16, and 21 post-onset and was treated with levofloxacin and methylprednisolone at a daily dosage of 10 mg/kg. The patient developed temporary necrotizing pneumonia, followed by complete recovery. **(A)** Bronchoscopy revealed an airway obstruction in the right upper lobe, characterized by a bronchial cast. **(B)** A bronchial cast observed during bronchoscopy. **(C)** A CT scan on day 40 of the illness course showed necrotizing pneumonia in the right upper lobe, with obstruction of the bronchi in the same region. **(D)** A follow-up CT scan, conducted 7 months after onset, demonstrated complete recovery.

**Figure 3:**
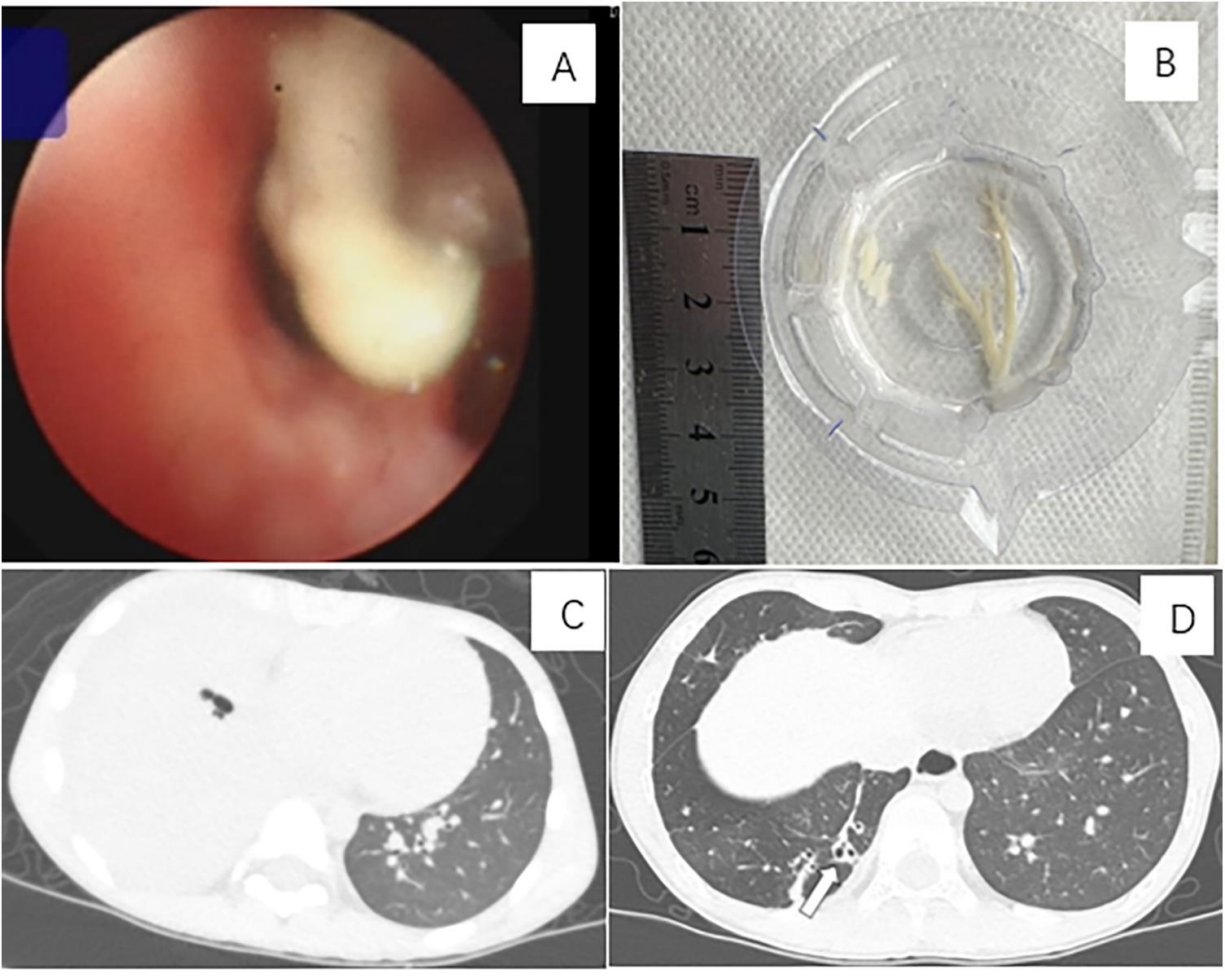
A case of plastic bronchitis associated with Mycoplasma pneumoniae infection resulted in persistent bronchiectasis. A 6-year-and-11-month-old boy was diagnosed with Mp infection. He underwent bronchoscopy intervention on days 17, 22, and 28 post-onset and was treated with levofloxacin and methylprednisolone at a daily dosage of 20 mg/kg. The patient experienced prolonged complications of bronchiectasis. **(A)** Bronchoscopy revealed an airway obstruction in the basal segment of the right lung, characterized by a bronchial cast. **(B)** A bronchial cast by bronchoscopy. **(C)** A CT scan on day 11 of the illness course showed consolidation in the basal segment of the right lung, with obstruction of the bronchi in the same region. **(D)** Nearly one year after the onset, a follow-up CT scan demonstrated bronchiectasis in the basal segment of the right lung.

Chest imaging of the 5 non-Mp cases revealed 1case of lung consolidation with pleural effusion, 2 with consolidation and atelectasis, and 2 with high-density pulmonary opacities. Following treatment, all chest imaging abnormalities resolved without long-term sequelae. Among the 81 patients with Mp, 25 developed pleural effusion, 24 suffered from atelectasis, 14 developed necrotizing pneumonia, 5 developed bronchiectasis, 3 had pulmonary interstitial emphysema, and 2 had pulmonary embolism during their illness. Following medical and bronchoscopy interventions, 65 children （the group of good outcome）exhibited complete or substantial resolution of pulmonary lesions without significant long-term sequelae observed on chest computed tomography (CT) scans (A selected case is illustrated in Figure 2). Long-term outcomes（the group of unclear outcomes） could not be assessed in 10 patients due to either lack of follow-up chest imaging or insufficient follow-up duration. 6 patients experienced long-term complications, including 1 who underwent surgical intervention（the group of adverse outcomes）, 3 with persistent atelectasis, 1 with both atelectasis and bronchiectasis, and 1 with persistent bronchiectasis (Figure 3). The patients in both the good-outcome and adverse-outcome groups were followed for a period ranging from six months to three years.

Radiographic manifestations and comorbidities of Mp-PB are listed in Table 2. Radiographic outcomes of the group of adverse outcomes are listed in Supplementary Table S1.

**Table 2:** Statistical analysis of demographics, symptoms, laboratory findings, radiographic complications and treatment between good-outcome group and adverse-outcomes group of Mp-PB.

Variables	Whole	Good-outcome group	Adverse-outcomes group	*P*-Value
	*n* = 81	(*n* = 65)	(*n* = 6)	
Demographic				
Sex (male)	53 (65.4)	43 (66.2)	5 (83.3)	0.65
Age (year)	6.9 (5.9–8.7)	6.9 (6.0–8.7)	5.5 (3.0–8.2)	0.25
Weight (kg)	21.00 ± 3.11	21.47 ± 2.87	18.68 ± 3.47	**0**.**05**
Height (cm)	114.85 ± 8.19	116.63 ± 7.52	105.67 ± 8.99	**0**.**01**
Symptoms				
Fever peak(°C)	39.70 ± 0.58	39.70 ± 0.57	39.70 ± 0.78	0.33
Cough	81 (100)	65 (100)	6 (100)	
Rash	16 (19.8)	11 (16.9)	2 (33.3)	0.30
Laboratory Results				
WBC (×10^9^/L)	7.02 (5.52–9.57)	6.89 (5.50–9.60)	7.57 (6.00–15.30)	0.37
CRP (mg/L)	18.0 (6.5–47.8)	17.3 (5.4–47.2)	35.2 (24.9–54.2)	0.17
	*n* = 80	*n* = 64		
PCT (ng/mL)	0.19 (0.10–0.37)	0.20 (0.10–0.37)	0.10 (0.05–0.58)	0.28
	*n* = 72	*n* = 59	*n* = 4	
IL-6 (pg/mL)	19.38 (10.00–62.85)	19.70 (10.50–61.40)	45.45 (1.27–66.94)	0.96
	*n* = 67	*n* = 55	*n* = 3	
LDH (U/L)	443.5 (323.5–644.7)	408.0 (307.2–550.8)	788.5 (629.3–1,034.5)	**0**.**01**
	*n* = 78	*n* = 62		
D-dimer (μg/mL)	0.92 (0.52–2.08)	0.81 (0.49–1.71)	2.20 (1.59–3.30)	**0**.**01**
	*n* = 77	*n* = 62		
Immunoglobulin E (IU/mL)	173.0 (62.8–423.1)	159.8 (56.8–374.6)	337.5 (31.5–950.6)	0.80
	*n* = 67	*n* = 55	*n* = 4	
Abnormal liver function	18 (22.2)	12 (18.5)	3 (50.0)	0.10
Resistance to Mp	39 (83.0)	30 (79.0)	4 (100)	0.57
	*n* = 47	*n* = 38	*n* = 4	
Radiographic complications				
Pleural effusion	25 (30.9)	18 (27.7)	4 (66.7)	0.07
Atelectasis	24 (29.6)	17 (26.2)	6 (100)	**0**.**00**
Necrotizing Pneumonia	14 (17.3)	14 (21.5)	4 (66.7)	**0**.**03**
Bronchiectasis	5 (6.2)	5 (7.7)	2 (22.3)	0.10
Pulmonary interstitial emphysema	3 (3.7)	3 (4.6)	0	1.00
Pulmonary embolism	2 (2.5)	2 (3.1)	1 (16.7)	0.24
Treatment				
Num-Bronchoscopy	2.00 (1.50–3.00)	2.0 (1.00–3.00)	3.50 (2.75–4.75)	**0**.**00**
Time-Bronchoscopy	10.0 (8.0–12.0)	10.0 (7.5–11.5)	12 (7.5–15.3)	0.35
Num-Methylprednisolone	79 (97.5)	63 (96.9)	6 (100)	1.00
Time-Methylprednisolone	7.0 (6.0–8.0)	7.0 (5.0–8.0)	6.0 (3.5–8.0)	0.26
	*n* = 79	*n* = 63		
Max-Methylprednisolone (mg/kg.d)	5.0 (3.0–10.0)	5.0 (3.0–6.0)	20.0 (4.9–20.0)	**0**.**01**
	*n* = 79	*n* = 63		
Doxycycline or levofloxacin	55 (67.9)	41 (63.1)	5 (83.3)	0.41
LMWH	24 (29.6)	14 (21.5)	6 (100)	**0**.**00**
IGIV	9 (11.1)	6 (9.2)	3 (50)	**0**.**02**
Thoracentesis	5 (6.2)	4 (6.2)	1 (16.7)	0.37
Oxygen	7 (8.6)	7 (10.8)	0	
Operation	1 (1.2)	0	1 (16.7)	

WBC, white blood cell count; CRP, C-reactive protein; PCT, procalcitonin; LDH, lactate dehydrogenase; Num-Bronchoscopy, number of bronchoscopy interventions; Time-Bronchoscopy, time to first bronchoscopy (days after symptom onset); Num-Methylprednisolone, number of administration with methylprednisolone; Time-Methylprednisolone, time of first administration with methylprednisolone (days after symptom onset); Max-Methylprednisolone, maximum daily dose of methylprednisolone; LMWH, low molecular weight heparin anticoagulation; IGIV, intravenous human immunoglobulin.

This table presents the overall data of 81 cases of MP-PB, including data for both the Good-outcome group and the Adverse-outcome group. For data pertaining to the group of unclear outcomes, please refer to the Supplementary Documents.

Parameters without noted sample sizes indicate no missing data, while sample sizes for parameters with missing data are explicitly noted.

Data are presented as mean ± standard deviation or interquartile range, depending on the distribution. Bold values denote *p* < 0.05.

### Demographics and symptoms in Mp-PB

All 81 patients with Mp-PB presented with symptoms such as fever and cough. No significant statistical differences were observed in age or gender distribution between the good and adverse outcomes groups. The height and weight in the Good-outcome group were significantly greater than those in the Adverse-outcomes group. In these groups, 11 (17%) and 2 (33%) cases, respectively, developed rashes, with no significant statistical difference.

### Laboratory results in Mp-PB

No significant differences were observed between the good and adverse outcomes groups in the following laboratory parameters: WBC (6.89 vs. 7.57 × 10^12^/L), CRP (17.3 vs. 35.7 mg/L), PCT (0.2 vs. 0.1 ng/mL), IL-6 (19.7 vs. 45.45 pg/mL), and IgE (159.8 vs. 337.5 IU/mL). However, the adverse outcomes group demonstrated significantly elevated LDH (408 vs. 788.5 U/L) and D-dimer (0.81 vs. 2.20 μg/mL). In the good and adverse outcomes groups, 12 (18%) and 3 (50%) cases, respectively, showed abnormal liver function, with no significant statistical difference observed between the groups.

### Drug treatment in Mp-PB

In the good-outcome group, 41 cases (62%) received second-line drugs (doxycycline or levofloxacin), compared to 5 cases (83%) in the adverse-outcomes group; however, this difference was not statistically significant. In the good-outcome group, 63 cases were administered with systemic glucocorticoids, whereas all children in the adverse-outcomes group received glucocorticoids. There was no significant difference in the timing of first glucocorticoid administration between the groups, with administration occurring on day 7 in the good-outcome group and on day 6 in the adverse-outcomes group. Children in the adverse-outcomes group received a significantly higher daily dose of methylprednisolone (20 mg/kg vs. 5 mg/kg) than those in the good-outcome group.

### Bronchoscopy interventions in Mp-PB

All 81 patients underwent bronchoscopy intervention. Of these, lavage and suction were performed in 50 cases, forceps removal in 30 cases, N-acetylcysteine lavage in 4 cases, and basket retrieval in 2 cases. No significant difference was observed in the timing of the initial bronchoscopy intervention between the good and adverse outcomes groups (10 vs. 12 days). However, the adverse-outcomes group required a significantly greater number of bronchoscopy interventions compared to the good-outcome group (3.5 vs. 2), a difference that was statistically significant.

### Ancillary treatments in Mp-PB

Low molecular weight heparin anticoagulation was administered to 14 patients in the good-outcome group, compared to all children in the adverse-outcomes group, a difference that was statistically significant. In the good-outcome group and the adverse-outcomes group, thoracentesis was performed in 4 and 1 cases, respectively. Human immunoglobulin therapy was given to 6 patients (9%) in the good-outcome group and 3 patients (50%) in the adverse-outcomes group. Oxygen therapy was administered to 7 patients in the good-outcome group, while none in the adverse-outcomes group received this treatment.

Statistical analysis of demographics, clinical symptoms, laboratory findings, radiographic complications and treatment of the good-outcome group and the adverse-outcomes group are presented in Table 2. Data of the group of unclear outcomes are presented in Supplementary Table S1.

## Discussion

Our study indicates that Mp is the most common pathogen associated with infection-related plastic bronchitis. Although the majority of cases related to Mp-PB exhibit favorable prognosis, a subset of pediatric patients may experience long-term complications. Additionally, elevated levels of LDH and D-dimer may serve as significant biomarkers for predicting outcomes in Mp-PB.

In 1902, Bettmann documented the first reported case of PB (11), and since then, the frequency of related case reports has gradually increased. The primary etiologies include post-congenital heart disease surgery (12, 13), asthma, cystic fibrosis, infections, lymphatic malformations, and sickle cell disease (14, 15), with non-infectious causes predominating (1). Recently, reports of infection-associated PB have increased, with pathogens such as Mp, influenza, adenovirus, and bocavirus identified as causative agents (5, 8). Although retrospective studies have summarized the clinical characteristics of infection-related PB, variations in pathogen distribution across different centers have been observed. Furthermore, treatment strategies, including the optimal timing and dosage of glucocorticoid therapy and the appropriate frequency of bronchoscopy interventions, remain controversial. Notably, no studies to date have specifically examined the outcomes of infection-related PB.

This study included 86 cases of infection-related plastic bronchitis. The analysis of pathogen distribution revealed that 94% of cases were attributed to Mp or co-infections involving Mp. Additional causative agents identified included influenza A virus, Hib combined with bocavirus, Sp, and co-infections of parainfluenza virus type 3 with human respiratory syncytial virus type 3. Mp is a well-recognized pathogen in pediatric pulmonary infections, contributing to approximately 8% of community-acquired pneumonia cases, as documented in the EPIC study (16). However, compared to other pathogens, Mp is more likely to result in PB. Numerous retrospective studies have identified Mp as a primary cause of infection-related PB (5, 8), suggesting that it may be a risk factor for the development of infection associated PB. The pathogenesis by which Mp more easily induces PB remains unclear. However, a study by Ma et al. suggests that it may be associated with the high expression of MUC5AC, MUC5B, and layilin induced by Mp (17). This study also found that the co-infection rate in PB cases due to Mp reached 36% (29/81), implying that co-infection may promote PB formation. In addition to Mp, influenza, particularly H1N1, has also been linked to PB, as first reported by Maki Hasegawa in 2009 (18), with subsequent case reports and retrospective analyses confirming this association (19). Additionally, bocavirus and adenovirus have been reported in conjunction with PB (8, 20), although cases involving Sp, Hib, Moraxella catarrhalis, and rhinovirus are rare (5). The etiology of infection associated PB demonstrates considerable variation across different centers. This disparity may be attributed, in part, to regional differences in the pathogen profiles of pulmonary infections and, in part, to the prevailing pathogens during the respective study periods. For example, the increased incidence of influenza-associated PB reported in 2010 was likely linked to the 2009 H1N1 influenza outbreak (18, 19). Similarly, the predominance of Mp in cases of infection-related PB reported between 2021 and 2023 may be associated with a contemporaneous epidemic of this pathogen (2–4, 7, 8, 21).

Pathogens play a crucial role in shaping the outcomes of PB. Non-Mp PB typically has a favorable prognosis following treatment. While Mp-related PB generally results in positive outcomes, a small subset of patients may experience severe or long-term complications. This study identified complications associated with Mp-PB, including pleural effusion, atelectasis, necrotizing pneumonia, bronchiectasis, interstitial lung air trapping, and pulmonary embolism. Post-treatment with medical and bronchoscopy interventions, most complications were resolved. Pleural effusion is a common complication of pediatric pulmonary infections, typically resolving with antibiotics, thoracentesis, and corticosteroid administration (9). Necrotizing pneumonia, although increasingly reported since its first documentation in 1994, remains rare. Initially attributed primarily to Sp and Sa (22), recent studies indicate a significant increase in cases involving Mp-related necrotizing pneumonia (23), with this study showing a 14% incidence in Mp-PB cases. Bronchiectasis, though uncommon as a sequela of pediatric lung infections, was observed in 5 cases, with 3 showing resolution on imaging following treatment, suggesting a certain degree of reversibility in pediatric bronchiectasis. This finding aligns with the research by Mills and colleagues, who reported a radiographic resolution rate of 40.1% in pediatric bronchiectasis (24). However, 2 cases exhibited persistent bronchiectasis. Previous studies have identified Mp as a cause of acquired bronchiectasis (25). Our research suggests that PB may be one of the mechanisms by which Mp leads to bronchiectasis. Of the 6 patients who experienced poor outcomes, 1 required surgical intervention due to encapsulated pleural effusion and delayed resolution of pulmonary infection. Additionally, 4 patients developed persistent atelectasis, which is likely attributable to incomplete or delayed clearance of bronchial casts, resulting in bronchial obstruction.

What factors can facilitate the early identification of pediatric patients with Mp-PB who are at risk for adverse outcomes? This study demonstrated that LDH and D-dimer levels were elevated in the adverse-outcomes group compared to the good-outcome group, indicating their potential as biomarkers for early identification of adverse outcomes in Mp-PB. LDH is a widely utilized clinical biomarker across various fields, including oncology and infectious diseases. Previous research has demonstrated that LDH levels can reflect the severity of Mp infections (26), help identify refractory Mp pneumonia, and predict the formation of plastic bronchitis (3, 4). D-dimer, although primarily used in clinical practice to diagnose venous thrombosis, has also been increasingly recognized as a biomarker for identifying and predicting severe Mp pneumonia and Mp-PB (3). In addition to LDH and D-dimer, there were no statistically significant differences in WBC, CRP, IL-6, or IgE levels between the good and adverse outcomes groups. However, CRP and IgE levels were notably higher in the adverse- outcomes group, suggesting that the lack of statistical significance may be due to an insufficient sample size. Previous studies have shown that CRP can help distinguish refractory Mp pneumonia (27). Nonetheless, whether CRP and IgE can predict the prognosis of Mp-PB requires further investigation with larger sample sizes. Other studies, such as that by Zhang have suggested that WBC, CRP, and IL-6 may aid in identifying refractory Mp pneumonia (28). However, the small sample size of this study may have led to the absence of statistical significance, necessitating further research with larger cohorts for validation.

In terms of treatment, the current approach to managing infection-related PB includes bronchoscopy intervention, antibiotic therapy, corticosteroid administration, and supportive care.

Bronchoscopy intervention remains the primary method for removing bronchial casts, with techniques such as lavage, forceps extraction, and endoscopic medication delivery being employed. Most bronchial casts can be effectively and promptly removed through these endoscopic procedures. Our study indicates that patients in the adverse-outcomes group underwent a greater number of bronchoscopy interventions compared to those in the good- outcome group, suggesting that bronchial casts in the adverse-outcomes group are more prone to recurrence or are more challenging to remove. However, despite the increased frequency of bronchoscopy interventions in these patients, this did not seem to significantly improve their prognosis. This finding serves as a reminder that while bronchoscopy intervention is a cornerstone in the treatment of PB, other therapeutic modalities are equally critical. These include the appropriate use of antimicrobial agents and timely, judicious administration of corticosteroids. Moreover, for cases involving recurrent, difficult-to-remove casts, or casts located in distal airways beyond the reach of the bronchoscopy, there is a need to explore new therapeutic approaches. Current efforts include the experimental use of fibrinolytic agents and other bronchoscopy techniques, although their efficacy remains uncertain at this time (29).

Regarding antibiotic therapy, the adverse-outcomes group had a higher utilization of second-line agents, particularly levofloxacin. The issue of macrolide-resistant Mp has become increasingly prevalent, especially in East Asia (30). Research by Chen and colleagues has revealed that the resistance rate of Mp reach 88.1% (31), with resistance primarily associated with mutations at sites such as A2063G, A2064G, and A2067G, with A2063G being the most common (21, 30, 31). The apply of new tetracyclines and quinolones in pediatric patients with drug-resistant Mp infection remains controversial due to safety concerns. In our cohort, 68% of patients with Mp-PB received second-line agents, with higher usage in the adverse outcomes group, possibly due to the 100% resistance rate in this subgroup.

All patients in the adverse-outcomes group received systemic glucocorticoid therapy, with higher doses administered compared to the good-outcome group, potentially reflecting a more pronounced inflammatory response. However, the efficacy of early or high-dose glucocorticoid therapy in preventing adverse outcomes remains uncertain.

Low molecular weight heparin was administered as prophylaxis in most cases, except for 2 pediatric patients with pulmonary embolism who required therapeutic anticoagulation. Human immunoglobulin support was more frequently utilized in the adverse- outcomes group. Interestingly, no children in the adverse-outcomes group received oxygen therapy, while more children in the good-outcome group did, indicating that oxygenation levels may not reliably predict long-term outcomes.

This study's retrospective nature and small sample size are limitations, and selection bias cannot be ruled out. Prospective studies with larger cohorts are needed to further clarify these findings. Additional limitations include the exclusive focus on A2063G resistance genes without broader drug sensitivity testing, the absence of pathological examination of bronchial casts, and the short follow-up period, which leaves the long-term outcomes of the adverse outcomes group uncertain.

Several key issues regarding infection-related PB remain unresolved, including early identification and intervention strategies, optimal treatment for long-standing, refractory casts, and the long-term prognosis of patients with sequelae. PB, as a complication of severe pulmonary infections, is a critical factor in the persistence of pulmonary sequelae and warrants early identification and appropriate management to improve outcomes and reduce the risk of chronic lung disease.

## Data Availability

The original contributions presented in the study are included in the article/Supplementary Material, further inquiries can be directed to the corresponding author.
