# Supplementary material for: Initial presentation, etiology and risk factors for adverse outcomes in infection-associated plastic bronchitis in children: a retrospective study
Source: Front Pediatr. 2026 Mar 13;14:1740626. doi: 10.3389/fped.2026.1740626 (PMC13021623; doi:10.3389/fped.2026.1740626)
Supplement: Supplementary file 1 [file Supplementaryfile1.docx]

FIGURE S1 A case of plastic bronchitis associated with influenza A infection


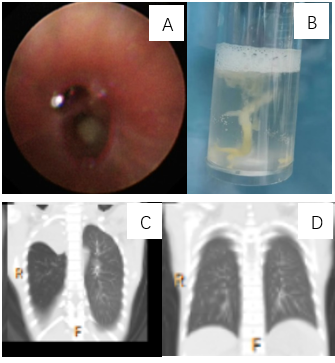


A 7-year-old girl was admitted to the hospital with a complaint of fever for 3 days, cough and wheezing for 2 days, and worsening dyspnea for 4 hours. She was diagnosed with an influenza A infection and underwent bronchoscopy on the fourth day after symptom onset.

**A**: Bronchoscopy identified an airway obstruction in the right upper lobe caused by a bronchial cast.

**B**: A bronchial cast was detected during bronchoscopy.

**C**: A CT scan performed on the third day of illness revealed consolidation and partial atelectasis in the right upper lobe.

**D**: A follow-up CT scan on day 9, after the bronchial cast was removed through bronchoscopy, showed complete resolution of the right upper lobe.

T Table S1 Statistical analysis of demographics, symptoms, laboratory findings, and treatment in group of Unclear-outcomes of Mp-PB.

|  | Unclear-outcomes group  (n=10) |
| --- | --- |
| **Demographic** |  |
| Sex (male) | 5 (50) |
| Age (year) | 7.1 (6.2-9.1) |
| Weight (Kg) | 21.66 ± 2.93 |
| Height (cm) | 117.04 ± 7.61 |
| **Symptoms** |  |
| Fever peak(℃) | 39.8±0.4 |
| Cough | 10 (100) |
| Rash | 3 (30) |
| **Laboratory Results** |  |
| WBC (×10^9^/L) | 7.5 (5.3-10.1) |
| CRP (mg/L) | 40.8 (2.9-95.8) |
| PCT (ng/mL) | 0.20 (0.10-0.38) |
| IL-6 (pg/mL) | 12.3 (1.3-96.0)  n=9 |
| LDH (U/L) | 514.5  (386.3-747.5) |
| D-dimer (μg/mL) | 1.65 (0.74-3.44)  n=9 |
| Immunoglobulin E (IU/mL) | 327.8  (100.0-496.6)  n=8 |
| Abnormal liver function | 3 (30) |
| Resistance to Mp | 5 (100)  n=5 |
| **Radiographic complications** |  |
| Pleural effusion | 3 (30) |
| Atelectasis | 1 (10) |
| necrotizing Pneumonia | 3 (30) |
| bronchiectasis | 1 (30) |
| Pulmonary interstitial emphysema | 0 |
| Pulmonary embolism | 1 (10) |
| **Treatment** |  |
| Num-BronchoscopyNum-B | 2 (2-3) |
| Time-BronchoscopyTime-B | 11 (8-12) |
| Num-MethylprednisoloneNum-M | 10 |
| Time-MethylprednisoloneTime-M | 7.5 (6-10.8) |
| Max-MethylprednisoloneMax-M | 5 (2.8-10) |
| Doxycycline or levofloxacinDoxycycline or levofloxacin, n(%) | 9 (90%) |
| LMWHLMWH | 4 (40%) |
| IGIVIg | 0 |
| ThoracentesisThoracentesis | 0 |
| OxygenOxygen | 0 |
| OperationOperation | 0 |
